# The effect of raloxifene augmentation in men and women with a schizophrenia spectrum disorder: a systematic review and meta-analysis

**DOI:** 10.1038/s41537-017-0043-3

**Published:** 2018-01-10

**Authors:** Janna de Boer, Merel Prikken, Wan U. Lei, Marieke Begemann, Iris Sommer

**Affiliations:** 1 Department of Psychiatry, University Medical Center Utrecht, Utrecht University & Brain Center Rudolf Magnus, Utrecht, The Netherlands; 2 Department of Neuroscience and Department of Psychiatry, University Medical Center Groningen, University of Groningen, Groningen, The Netherlands; 3 Department of Biological and Medical Psychology, University of Bergen, Bergen, Norway

## Abstract

Recognizing the robust sex differences in schizophrenia prevalence, the selective estrogen receptor modulator (SERM) raloxifene is a likely candidate for augmentation therapy in this disorder. Therefore, a systematic search was performed using PubMed (Medline), Embase, PsychInfo, and Cochrane Database of Systematic Reviews. Randomized controlled trials investigating the effect of raloxifene in schizophrenia spectrum disorders were included in the quantitative analyses. Outcome measures were psychotic symptom severity, depression, and cognition. Meta-analyses were performed using Comprehensive Meta-Analysis software. A random-effects model was used to compute overall weighted effect sizes in Hedges’ *g*. Nine studies were included, investigating 561 patients with a schizophrenia spectrum disorder. Raloxifene was superior to placebo in improving total symptom severity (*N* = 482; Hedge’s *g = *.57, *p* = 0.009), as well as positive (*N* = 561; Hedge’s *g = *0.32, *p* = 0.02), negative (*N* = 561; Hedge’s *g = *0.40, *p* = 0.02), and general (*N* = 526; Hedge’s *g = *0.46, *p* = 0.01) subscales, as measured by the Positive and Negative Syndrome Scale. No significant effects were found for comorbid depression and cognitive functioning. Altogether, these results confirm the potential of raloxifene augmentation in the treatment of schizophrenia.

## Introduction

In schizophrenia, robust sex differences exist with an incidence risk ratio of 1.4 for men as compared to women.^1^ In addition, age of onset is significantly lower in men,^2^ while women (but not men) show a second incidence peak after the age of 50.^3^ Premenopausal women experience a more favorable course than men, with lower psychotic and lower negative symptoms, better cognitive and social functioning,^3^ and approximately 50% less hospitalizations.^4^ The most likely explanation for these sex differences is that estrogens have a protective role in the pathophysiology of schizophrenia.^3^ Indeed, female patients with schizophrenia have more severe symptoms in the low estrogen phase of their menstrual cycle.^5^ Studies controlling for estrogen plasma levels also demonstrated a negative correlation between 17β-estradiol levels and severity of schizophrenia symptoms.^6^ Furthermore, higher estrogen levels were strongly correlated with better cognitive performance in women with schizophrenia.^7^ This potentially ameliorating role of estrogens on symptom severity provides an important lead for a new treatment strategy for patients with schizophrenia.

Several trials have been conducted to evaluate the clinical potential of estrogen augmentation therapy in premenopausal women with schizophrenia. In a meta-analysis of five randomized controlled trials (RCTs) we found a significant weighted effect size of 0.66 (95% confidence interval 0.21 to 1.11) for the efficacy of estrogens on total symptom severity in women with schizophrenia.^8^ Importantly, estrogens were superior to placebo in improving positive symptoms and negative symptoms (Hedges’ *g* 0.54 and 0.34, respectively). These promising results are not easily translated into daily practice. While the participants experienced improvement with this treatment, all RCTs provided estrogens for 4 to 8 weeks only.^8^ Long-term use of estrogen is not safe as it has considerable side effects on the sex organs.^9^ Furthermore, estrogen augmentation is not indicated for men with schizophrenia, as estrogens have feminizing effects. Interestingly, selective estrogen receptor modulators (SERMs) do not carry these side effects, as they have agonistic action on estrogen receptors in the brain and bones, but not in the sex organs. SERMs such as raloxifene and tamoxifen could, therefore, have therapeutic benefits in schizophrenia patients of both sexes without being hazardous to gynecological tissues or having feminizing effects. Currently, raloxifene is the only SERM that is approved for long-term treatment. In the last 7 years, several studies have been carried out to assess the potential effect of raloxifene on symptoms and comorbidities in schizophrenia.^9,10^ This paper provides a quantitative systematic review investigating efficacy of this new therapy for positive, negative, and general symptoms of schizophrenia. In addition, we examine its effects on depression and cognition.

## Results

**Fig. 1:**
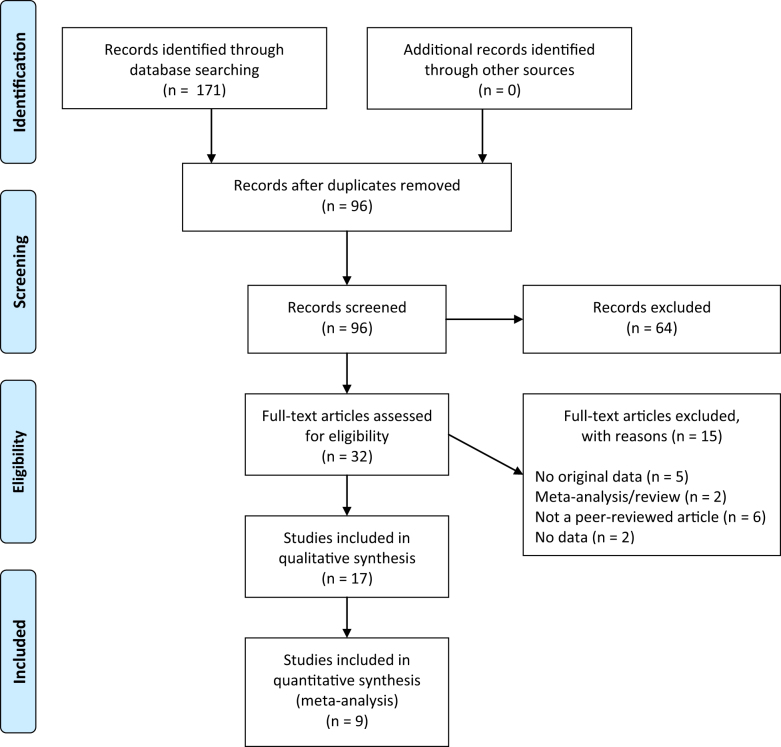
Flow diagram of the search

A flow diagram of the literature search is depicted Fig. 1. After screening on title and abstract, the search yielded 32 articles on raloxifene use in psychosis. After full-text reading seventeen articles remained of which eight articles were case-reports,^11–18^ which are described in Table S1. Nine studies were included in the meta-analyses (see Table 1 for descriptive information). Taken these studies together, the efficacy of raloxifene versus placebo was assessed in a total of 561 patients.^19–27^

**Table 1:** Main characteristics of studies included in quantitative assessment

Study	*N*	Group	Mean age in years ± SD		Illness duration/age at onset mean years ± SD		Daily dose (mg)	Treatment duration (weeks)
			Raloxifene	Placebo	Raloxifene	Placebo		
Kulkarni et al. (2010)^a^	21	F (post)	53.3 ± 8.0	50.9 ± 4.2	25.7 ± 10.1/ -	11.6 ± 6.5 / -	120	12
Kulkarni et al. (2010)^a^	14	F (post)	54.6 ± 4.6	50.9 ± 4.2	24.9 ± 11.5 / -	11.6 ± 6.5 / -	60	12
Usall et al. (2011)/Huerta-Ramos et al. (2014)^b^	33	F (post)	60.1 ± 6.41	62.7 ± 4.54	27.7 ± 6.97 / -	25.2 ± 11.12 / -	60	12
Kianimehr et al. (2014)	46	F (post)	62.0 ± 4.49	60.44 ± 5.28	17.2 ± 12.03 / 35.0 ± 11.69	13.6 ± 12.41 / 29.4 ± 8.57	120^c^	8
Khodaie-Ardakani et al. (2015)	42	M	32.4 ± 7.8	31.4 ± 5.9	8.0 ± 3.83 / -	7.4 ± 5.91 / -	120^c^	8
Weickert et al. (2015)	79	M, F (pre + post)	37.4 ± 7.3	34.0 ± 8.4	13.4 ± 7.5 / 24.1 ± 4.8	12.2 ± 7.2 / 22.1 ± 6.3	120	6
Kulkarni et al. (2016)	56	F (per + post)	52.9 ± 8.07	53.1 ± 7.43	- / 27.9 ± 11.60	- / 28.6 ± 12.34	120	12
Usall et al. (2016)	70	F (post)	62.0 ± 9.39	61.3 ± 10.41	- / 26.3 ± 8.64	- / 27.0 ± 11.37	60	24
Weiser et al. (2017)	200	F (post)	55.8 ± 4.7	56.6 ± 4.6	- / 32.0 ± 9.5	- / 31.1 ± 8.6	120	16

*N* sample size, *SD* standard deviation, *F* female, *post* postmenopausal, *pre* premenopausal, *per* perimenopausal, *M* male, *mg* milligram

^a^The study by Kulkarni et al. (2010) administered two different dosages and, therefore, two effect sizes were extracted from each report. The reported *N* per effect size is number of patients in intervention group, plus a proportional amount of the number of patients in the placebo group

^b^The papers by Usall et al. (2011) and Huerta-Ramos et al. (2014) are reported as one sample in this table, since they both report on the same sample and thus main characteristics are the same. N.B. Usall et al. (2016) reports on a different sample and is, therefore, presented separately

^c^All participants received risperidon 6 mg in addition to raloxifene or placebo

Of the nine RCTs, one study had a crossover design. Since the authors found a significant carryover effect, only the results before crossover were used in the meta-analysis.^26^ In all studies, patients were treated with a stable dose of antipsychotics and no relevant dose changes were allowed during the trials.

### Primary outcome measure: symptoms severity

**Fig. 2:**
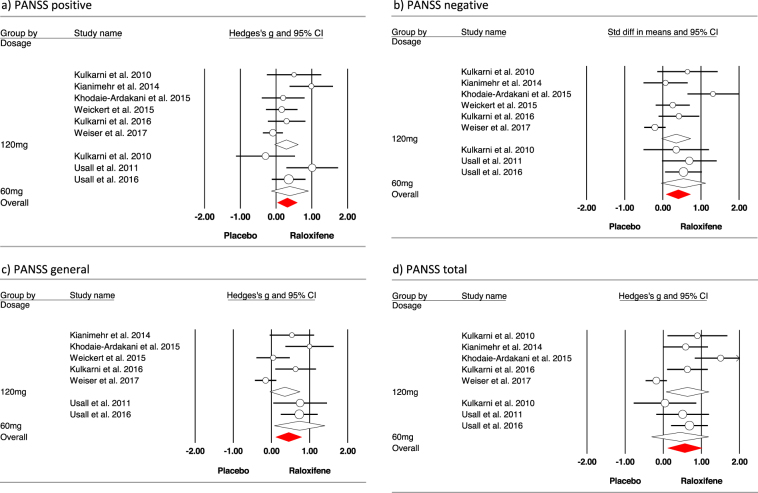
Meta-analysis of the effect of raloxifene addition symptom severity, as measured with PANSS. Studies are grouped by dosage

Results for primary study outcome measures are depicted in Fig. 2 and Table 2. Moderate, but significant effect sizes were found for PANSS total, as well as the positive, negative, and general subscales.^19–27^ Subsequently, subgroup analyses were performed for 60 and 120 mg dosages (see Fig. 2). Between group analyses revealed that effect sizes did not differ between these dosages (all *p*’s > 0.30). Meta-regression showed that treatment duration was not related to effect sizes found in the studies (all *p*’s > 0.52).

**Table 2:** Statistical results regarding all outcome measures

Outcome measure	Studies *N*	Subjects *N*	Hedges’ *g* (95% CI)	*p*-value	*I* ^2^	Q-value (*p*-value)	Egger’s test
PANSS^a^							
** Positive**	**9**	**561**	**0.32 (0.05–0.59)**	**0.02**	**54.24**	**17.48 (0.03)**	**0.11**
** Negative**	**9**	**561**	**0.40 (0.08–0.72)**	**0.02**	**67.23**	**24.42 (0.002)**	**0.03**
** General**	**7**	**526**	**0.46 (0.01–0.82)**	**0.01**	**74.01**	**23.08 (0.001)**	**0.005**
** Total**	**8**	**482**	**0.57 (0.41–0.99)**	**0.009**	**77.51**	**31.12 (<0.001)**	**0.05**
Depression	2	135	0.14 (−0.20–0.47)	0.43	N/A	0.33 (0.57)	N/A
Cognition							
Attention and working memory	4	352	−0.01 (−0.28–0.26)	0.92	27.25	4.12 (0.25)	0.66
Executive functioning	2	221	0.03 (−0.23–0.29)	0.83	N/A	0.42 (0.52)	N/A
Memory	4	361	0.12 (−0.11–0.35)	0.31	13.22	3.46 (0.33)	0.16
Psychomotor speed	3	303	0.28 (−0.26–0.81)	0.31	74.68	7.90 (0.02)	0.28
Verbal fluency	4	361	0.06 (−0.24–0.35)	0.71	41.58	5.14 (0.16)	0.83
Global cognitive functioning	2	256	−0.13 (−0.37–0.11)	0.30	N/A	0.22 (0.64)	N/A

*N* number, *PANSS* Positive and Negative Syndrome Scale. Significant effect sizes in bold. *N/A* not applicable

^a^Not all studies reported all PANSS scales, therefore, the number of studies varies between subdomains

Heterogeneity was high for all PANSS subscales, see Table 2. Egger’s tests were significant for negative, general and total PANSS scores. However, there were no significant outliers in the data. Visual inspection of the funnel plots suggests a slight under publication of negative results for these three outcome measures. However, the asymmetry in the funnel plots can also be explained by the limited number of studies for each outcome measure.

### Secondary outcome measures: depression and cognition

No significant effects were found on depression^20,26^ or cognitive outcome measures (see Table 2).^20,23,26,27^ Since the meta-analyses for depression, executive functioning, and global cognitive functioning included only two studies each, Egger’s test and *I*^2^ could not be calculated. Heterogeneity was low for attention and working memory, memory, and verbal fluency analyses.

## Discussion

This meta-analysis provides a systematic overview of current literature regarding efficacy of raloxifene as an augmentation to antipsychotic medication in schizophrenia. Nine studies could be included that all compared raloxifene to placebo in a double-blind randomized controlled design. We found moderate, but significant positive effects of raloxifene on total symptom severity, as well as on positive, negative, and general PANSS subscales. Dosage or treatment duration did not influence these effects. Only few studies assessed the effect of raloxifene in schizophrenia patients on depression and cognition. We performed meta-analyses on the studies that also assessed these outcome measures, but found no significant benefit from raloxifene.

This finding is largely in agreement with the results from our previous meta-analysis on estrogen addition for patients with schizophrenia, which also showed a significant effect of moderate size on positive and negative symptoms.^8^ Furthermore, the results confirm a previous meta-analysis on raloxifene use in postmenopausal women,^10^ and extend these results to a larger population. In contrast to estrogen, raloxifene has been well tolerated in the treatment of breast cancer and osteoporosis for periods of years.^28,29^ Furthermore, as opposed to estrogens, raloxifene can also be provided to men without affecting primary or secondary sex organs.^22,26^ Although the risk for endometrium and other forms of cancer appears to be low with raloxifene, this drug does carry an increased risk for venous thrombo-embolic events,^30^ comparable to the associated risk of contraceptives.

The greatest strength of the present study is that it provides an up-to-date, quantitative and qualitative overview of the literature regarding the efficacy of raloxifene augmentation in schizophrenia. A recent meta-analysis by Wang and colleagues on the same topic showed beneficial effects of raloxifene on psychotic symptoms in postmenopausal women with schizophrenia.^10^ However, studies investigating men and premenopausal women with schizophrenia were excluded from this meta-analyis. Therefore, although finding similar results, the present study, provides a more thorough overview of the current literature.

Additionally, the current meta-analysis provides insight into the effects of raloxifene on cognitive functioning in patients. Previous research has shown that raloxifene reduces the risk of cognitive decline in healthy postmenopausal women,^31^ which suggests that raloxifene has a protective effect on cognitive functioning. Although several RCT’s and case-reports reported beneficial effects on cognition,^12–14,16,18,20,23,26,27^ our meta-analytic results could not confirm these findings. However, given the small number of studies that could be included in the analyses regarding cognitive functioning, our results do not exclude the possibility that in some cases raloxifene *can* improve cognitive functioning in patients with schizophrenia. It could be the case that cognitive functioning improves only after longer duration of treatment, since previously it was demonstrated that raloxifene reduced cognitive decline after 3 years of treatment with raloxifene.^31^ Symptom severity could also be a confound in this matter, since the studies that investigated effects on cognitive functioning included patients that were more severely ill. It could be that greater symptom severity interferes with improvements in cognitive functioning, as was suggested in a previous discussion on this topic.^32^ More studies on this topic are necessary to provide reliable conclusions.

In sum, raloxifene is consistently shown to be effective as an augmentation to antipsychotic medication to ameliorate psychotic symptoms of schizophrenia. As this medication can be used by men and women for longer time periods, it appears to be a valuable addition to current therapeutic options.

## Methods

### Literature search

This meta-analysis was performed according to the Preferred Reporting for Systematic Reviews and Meta-analysis (PRISMA) Statement.^33^ The literature search was conducted by two independent researchers (C.L. and M.P.) using Pubmed (Medline), Embase, Cochrane Database of Systematic Reviews, and Psychinfo. Combinations of the following search terms were used: “raloxifene”, “evista” or “SERM” and “schizophrenia”, “psychosis”, “psychotic”, “schizoaffective”, or “schizophreniform”. The search had no year and language restrictions. See Table S2 for an example search string. The search cutoff date was 10 October 2017. Reference lists of the included studies were searched for cross-references. After independent screening was performed by M.P. and C.L., consensus about the included studies was reached between all authors.

### Inclusion criteria

Articles were included when the following inclusion criteria were met: (1) randomized, double-blind placebo-controlled trials (used for quantitative synthesis) or case-reports (used for qualitative synthesis) that assessed the effect of raloxifene on one of our outcome measures; (2) included patients with schizophrenia spectrum disorder (schizophrenia, schizoaffective disorder, schizophreniform disorder or psychotic disorder not otherwise specified), according to the diagnostic criteria of the Diagnostic and Statistical Manual of Mental Disorders (DSM-III, DSM-III-R, DSM-IV, DSM-IV-TR, DSM-5)^34,35^, or the International Classification of Diseases (ICD-9 or ICD-10); (3) studies were published in a peer-reviewed journal. For two studies that included the same patient sample, outcome measures that were similar were included in the analysis only once.^23,24^ Risk of bias was assessed independently by J.B. and M.P. using the Cochrane Risk of Bias tool for RCTs (Table S3).^36^

### Outcome measures

The primary outcome measure was psychotic symptom severity, measured with the Positive and Negative Syndrome Scale (PANSS).^37^ Secondary outcome measures were cognitive functioning (for domains and included tests, see Table S4) and depressive symptoms (assessed by the Montgomery-Asberg Depression Rating Scale (MADRS)^38^ or Depression Anxiety and Stress Scale (DASS).^39^

### Statistics

Comprehensive meta-analysis (CMA) software version 2.0 was used to perform all analyses, using a random-effects model.^40^ For every individual study, Hedges’ *g* was calculated for each outcome measure. To obtain this effect size, per treatment arm, mean differences in change scores (end of treatment minus baseline) and standard deviations (SD)) or pre- and post-means ( + SD) were used. To avoid overestimation of the true effect sizes caused by the pre-post treatment correlation,^41^ change scores were preferred. When these values were not reported, we used exact *F*-, *t*-, or *p*-values. All effect sizes were calculated twice independently from the original articles to check for errors.

Studies were combined in meta-analyses to calculate a mean weighted effect size for each outcome measure, using a random-effects model. To investigate whether studies could be taken together to share a common population effect size, the *Q*-value and *I*^2^-statistic were evaluated for each analysis. The *Q*-statistic tests the existence of heterogeneity, and displays a chi-square distribution with k-1 degrees of freedom (*k* = number of studies), where *Q*-values higher than the degrees of freedom indicate significant between-studies variability. *I*^2^ reflects which proportion of the observed variance reflects differences in true effect sizes, rather than sampling error, ranging from 0 to 100%. Values of 25%, 50%, and 75% can be interpreted as low, moderate, and high, respectively.^42^

Additionally, funnel plots were inspected for asymmetry in order to check for publication bias. Potential asymmetry was tested with Egger’s test, using a significance level of *α* = 0.05 (2-tailed). Effect sizes with a *p*-value smaller than 0.05 were considered statistically significant. Effect sizes were interpreted according to the guidelines by Cohen, with an effect size of 0.20 indicating a small effect, 0.50 a medium and over 0.80 a large effect.^43^

As in all papers either a dosage of 60 mg or 120 mg raloxifene was administered, a subgroup analysis was performed based on this categorization. This was done for PANSS outcomes only, as the amount of papers that reported depressive symptoms or cognitive functioning as an outcome measure was insufficient to perform this analysis. Furthermore, to assess the effect of treatment duration, this variable was used as a regressor in additional analyses.

### Data-availability

The authors declare that the main data supporting the findings of this study are available within the article and its Supplementary files. Since this is a meta-analysis no primary data were collected during this study. Additional data are available from the corresponding author upon request.

## Electronic supplementary material

Supplementary Material
