# Supplementary material for: The effect of raloxifene augmentation in men and women with a schizophrenia spectrum disorder: a systematic review and meta-analysis
Source: NPJ Schizophr. 2018 Jan 10;4:1. doi: 10.1038/s41537-017-0043-3 (PMC5762671; doi:10.1038/s41537-017-0043-3)
Supplement: Supplementary file 1 — Supplementary Material [file 41537_2017_43_MOESM1_ESM.docx]

**Supplementary Material**

**Table S1 : Main characteristics and results of case-reports**

| **Study** | **N** | **Age (years)** | **Group** | **Daily dose (mg)** | **Treatment duration at publication**  **(months)** | **Side effects** | **Outcome** |
| --- | --- | --- | --- | --- | --- | --- | --- |
| Wong et al. 2003 | 6 | Mean  57.1 | F (post) | N/R | 2, 2^a^ | N/R | Improved cognition, though no significant difference with placebo. |
| Kulkarni et al. 2008 | 1 | 57 | F (post) | 120 | 3 | N/R | Improved cognitive functioning, especially verbal memory and psychomotor speed. |
| Shivakumar et al. 2012 | 1 | 17 | F (pre) | 120, 60^b^ | 4 | Menstrual blood clots, with normal clotting times | Reduction in restlessness, agitation, and hallucinatory behavior. Improved attention span and social interactions. |
| Sharma et al. 2012 | 1 | 29 | F (pre) | 60, 120^c^ | 7 | N/R | Improved socio- occupational functioning and reduction in symptom severity |
| Dhandapani et al. 2013 | 1 | 47 | F (pre) | 60 | 6 | N/R | Strong improvement in negative symptoms; became asymptomatic. |
| Tharoor & Goyal 2015 | 1 | 53 | F (post) | 60, 120^c^ | 6 | None | Patient became asymptomatic. |
| Huerta-Ramos et al. 2015 | 1 | 61 | F (post) | 60 | 6, 3^d^ | None | Improvement in symptoms and executive functioning. |
| Grigg et al. 2016 | 1 | 44 | F (pre) | 120 | 3 | None | Improvement in psychotic symptoms, mood and cognition. Menstrual cycle normalized during treatment. |

Table Legend: N = sample size, N/R = not reported, F = female, post = postmenopausal, pre = premenopausal, mg = milligram.

^a^ = Patients received 8 weeks of raloxifene and 8 weeks of placebo, and were randomized to either starting with raloxifene or starting with placebo.

^b^ = The patient received initial treatment with raloxifene 120mg, which was reduced to 60mg after 8 weeks. Clinical improvements remained after the dose reduction.

^c^ = The patient received initial treatment with raloxifene 60mg, which was increased to 120mg after 1 week of treatment.

^d^ = The patient received raloxifene for 6 months, after which treatment was withdrawn for 3 months. Raloxifene was restarted due to worsening of symptoms and was followed for another 3 months up to publication.

**Table S2: Systematic search strategy**

| **PUBMED** |
| --- |
| **#1: Domain**  **((((schizophren*[Title/Abstract] OR schizoaffective[Title/Abstract] OR psychosis[Title/Abstract] OR psychotic*[Title/Abstract] OR schizophreniform[Title/Abstract])) OR psychotic disorders[MeSH Terms]))** |
| **#2: Determinant**  **((((raloxifene[Title/Abstract] OR evista[Title/Abstract] OR selective estrogen receptor modulator*[Title/Abstract] OR SERM*[Title/Abstract])) OR raloxifene hydrochloride[MeSH Terms]) OR selective estrogen receptor modulators[MeSH Terms])** |
| **#3: #1 AND #2** |

**Table S3: Risk of bias assessment**

| **Study** | **Random allocation (selection bias)** | **Allocation concealment (selection bias)** | **Double blind setting (performance bias)** | **Blinding of outcome assessment (detection bias)** | **Incomplete outcome data (attrition bias)** | **Selective reporting (reporting bias)** | **Funding** |
| --- | --- | --- | --- | --- | --- | --- | --- |
| Kulkarni et al. 2010 | + | U | + | + | + | - | - |
| Usall et al. 2011 | + | + | + | + | + | + | + |
| Huerta-Ramos et al. 2014 | + | + | + | + | U | + | + |
| Kianimehr et al. 2014 | U | U | + | + | - | + | + |
| Weickert et al. 2015 | + | + | + | + | + | + | + |
| Khodaie-Ardakani et al. 2015 | + | + | + | + | U | + | + |
| Kulkarni et al. 2016 | + | + | + | + | + | + | + |
| Usall et al. 2016 | + | + | + | + | + | - | + |
| Weiser et al. 2017 | U | U | U | U | + | + | + |
| Table Legend: + = low risk of bias, - = high risk of bias, U = unclear risk of bias , n.a. = not applicable. | | | | | | | |

**Table S4: Overview of cognitive domains and included tests**

| **Attention and working memory** | **Executive functioning** | **Memory** |
| --- | --- | --- |
| WAIS-III Letter Number sequencing  RBANS attention  BACS digit sequencing | BACS tower of london  STROOP words and colors | WMS-R logical memory II  RBANS delayed memory  BACS verbal memory  TAVEC long-term memory |
| **Psychomotor speed** | **Verbal Fluency** | **Global cognitive functioning** |
| Trail Making Test A  BACS token motor task | COWAT  RBANS language  BACS verbal fluency  Semantic fluency test (animals) | RBANS total score  BACS composite |
